# Accumulation of Anthocyanins through Overexpression of AtPAP1 in *Solanum nigrum* Lin. (Black Nightshade)

**DOI:** 10.3390/biom10020277

**Published:** 2020-02-11

**Authors:** Saophea Chhon, Jin Jeon, Joonyup Kim, Sang Un Park

**Affiliations:** 1 Department of Crop Science, Chungnam National University, 99 Daehak-ro, Yuseong-gu, Daejeon 34134, Koreajeonjin519@cnu.ac.kr (J.J.); 2 Department of Horticultural Science, Chungnam National University, 99, Daehak-Ro, Yuseong-gu, Daejeon 34134, Korea

**Keywords:** Anthocyanin, *Solanum nigrum* L., flavonoid biosynthesis, AtPAP1

## Abstract

Black nightshade (*Solanum nigrum*) belongs to the *Solanaceae* family and is used as a medicinal herb with health benefits. It has been reported that the black nightshade plant contains various phytochemicals that are associated with antitumor activities. Here we employed a genetic approach to study the effects of overexpression of *Arabidopsis thaliana* production of anthocyanin pigment 1 (AtPAP1) in black nightshade. Ectopic expression of AtPAP1 resulted in enhanced accumulation of anthocyanin pigments in vegetative and reproductive tissues of the transgenic plants. Analysis of anthocyanin revealed that delphinidin 3-O-rutinoside-5-O-glucoside, delphinidin 3,5-O-diglucoside, delphinidin 3-O-rutinoside, petunidin 3-O-rutinoside (*cis*-*p*-coumaroyl)-5-O-glucoside, petunidin 3-(feruloyl)-rutinoside-5-glucoside, and malvidin 3-(feruloyl)-rutinoside-5-glucoside are highly induced in the leaves of AtPAP1 overexpression lines. Furthermore, ectopic expression of AtPAP1 evoked expression of early and late biosynthetic genes of the general phenylpropanoid and flavonoid pathways that include phenylalanine ammonia-lyase (*PAL*), cinnamate-4-hydroxylase (*C4H*), 4-coumarate CoA ligase (*4CL*), chalcone isomerase (*CHI*), and quinate hydroxycinnamoyl transferase (*HCT*), which suggests these genes might be transcriptional targets of AtPAP1 in black nightshade. Concomitantly, the total content of anthocyanin in the transgenic black nightshade plants was higher compared to the control plants, which supports phenotypic changes in color. Our data demonstrate that a major anthocyanin biosynthetic regulator, AtPAP1, can induce accumulation of anthocyanins in the heterologous system of black nightshade through the conserved flavonoid biosynthesis pathway in plants.

## 1. Introduction

Black nightshade (*Solanum nigrum*) is widely used as a leafy vegetable, fruit and source of various therapeutic drugs. Consumption of the leaves and fruits as food is widespread in Africa and Southeast Asia. As a whole plant, the black nightshade has been used as a folk medicine in Asia to treat inflammation, edema, and mastitis. Phytochemical screening of the crude extracts from the plant revealed the presence of secondary metabolites such as alkaloids, glycoproteins, flavonoids, polyphenols, and triterpenoids [1,2]. Although a wide range of bioactivities including anti-inflammatory, antioxidant, antinociceptive, antipyretic, antitumor, antiulcerogenic, cancer chemopreventive, hepatoprotective, and immunomodulatory effects have been reported, the roles of anthocyanins associated with these activities for the black nightshade were not fully determined. Anthocyanins are flavonoids linked to the pigmentation of plant flowers and fruit that ranges from red to blue. They also attract pollinators and animals for pollination and seed dispersal and defend plants against various biotic and abiotic stresses [3]. Anthocyanins also play a positive role in the reduction of many chronic diseases that result from oxidative stress [4]. The biosynthesis of anthocyanins is regulated by activities of many enzymes involved in the general phenylpropanoid pathway, which can be divided into two groups [5]. The upstream biosynthesis genes, chalcone synthase (*CHS*), chalcone isomerase (*CHI*), and flavone 3-hydroxylase (*F3H*), are the early biosynthetic genes (EBGs) common to the biosynthesis of all downstream flavonoids. The late biosynthesis genes (LBGs) such *as flavonoid 3′-hydroxylase* (*F3′H*), flavonoid 3′,5′-hydroxylase (*F3′5′H*), dihydroflavonol 4-reductase (*DFR*), anthocyanidin synthase/leucoanthocyanidin dioxygenase (*ANS*/*LDOX*), and flavonoid 3-O-glucosyltransferase (*UFGT*) are responsible for the biosynthesis of specific classes of flavonoids, including anthocyanins.

Previous studies have demonstrated that gene expression for the EBGs and the content of anthocyanins are not consistently correlated, and vary depending on the plants species [6]. On the contrary, it has been shown that there appears to be a consistent positive correlation between expression levels of the LBGs and anthocyanin levels in several *Solanaceae* plants. Gene expression of the EBGs (e.g., *CHS*, *CHI*, *F3H*), *F3′H*, and flavanol synthase (*FLS*) are shown to be regulated by R2R3 type MYB transcription factors. In regard to the LBGs including *DFR*, *LDOX*, and UDP-glucose: flavonoid 3-O-glucosyl transferase (*UF3GT*), it has been shown that expression of these genes are coordinately regulated by the MYB-bHLH-WD40 (MBW) transcription factor complex, consisting of R2R3 type MYB activators, bHLH activators, and the WD-repeat protein transparent testa glabara 1 (TTG1) [7,8,9].

*Arabidopsis* AtPAP1 encodes an MYB75 transcription factor, which is crucial to the biosynthesis of anthocyanins in *Arabidopsis*. Both knockout and knockdown mutants of AtPAP1 display deficiencies in anthocyanin pigmentation in seedlings, while ectopic expression of AtPAP1 overproduces anthocyanins throughout the plant (e.g., roots, flowers) resulting from coordinated up-regulation of anthocyanin biosynthesis genes [10]. Transient transactivation assay in a combination with bioinformatics assay uncovered that transcriptional activation of anthocyanin biosynthesis genes is mediated by AtPAP1 that targets a common *cis*-regulatory element present in the upstream region of the 5′-UTR of biosynthesis genes. The AtPAP1 binds to the 10 bp CCACG-containing PAP1 *cis*-regulatory element in the promoter regions of these anthocyanin biosynthesis-related genes (preferentially LBGs). In addition, light is essential for the transcriptional activation of AtPAP1 [11]. This light-dependent transcriptional activation of AtPAP1 is mediated by the leucine-zipper transcription factor HY5 that directly binds to G- and ACE-boxes in the promoter region of AtPAP1, which acts as a downstream component of phytochrome (PHY), cryptochrome (CRY), and UV-B (UVR8) photoreceptors [12]. Further, this light-dependent anthocyanin biosynthesis is positively regulated by sugar and cytokinin and negatively regulated by ethylene [13] and nitrogen deficiency [14]. In addition, it has been demonstrated that AtPAP1 activity is negatively regulated by repressor protein associated with the R3 type MYB-related protein, MYBL2 [8] and miR156-targeted squamosa lateral organ promoter binding protein-like 9 (SPL9) that destabilizes the MYB–bHLH–WD40 complex [15].

Here we utilized a genetic approach to study the effects of constitutive expression of AtPAP1 in black nightshade. Constitutive expression of AtPAP1 led to enhanced accumulation of anthocyanins in black nightshade. Using liquid chromatography-mass spectrometry (LC/MS/MS) six anthocyanins were identified in the AtPAP1 overexpression lines. Total anthocyanins analysis measured by spectrophotometry revealed that the transgenic plants contained a higher amount of anthocyanins compared with the control plants. In addition, ectopic expression of *Arabidopsis* AtPAP1 up-regulated several genes that are possibly involved in the biosynthesis of anthocyanins in black nightshade. Our results demonstrate that a major biosynthetic regulator can induce enhanced accumulation of anthocyanins through the conserved flavonoid biosynthesis pathways in black nightshade.

## 2. Materials and Methods

### 2.1. Identification of Phenylpropanoid Biosynthesis Genes in Black Nightshade

Genes involved in phenylpropanoid biosynthesis in the black nightshade (*S. nigrum*) were identified from RNA-seq data. The sequences of transcripts that correspond to the functional annotation available in the *Solanaceae* database were selected. In addition, the phenylpropanoid biosynthetic genes of *Arabidopsis* obtained from TAIR (https://www.arabidopsis.org/) were used as queries to search for homologous sequences in the *S. nigrum* transcriptome database. The deduced amino acid sequences of the retrieved genes were further analyzed for homology using BLAST in the NCBI GenBank database (https://blast.ncbi.nlm.nih.gov/Blast.cgi). The genes with a maximum identity were used for the further transcriptional study [16,17].

### 2.2. HPLC Analysis of Anthocyanin Content

Leaf samples were harvested and freeze-dried at −80 °C for 3 days. The freeze-dried samples were pulverized using a mortar and pestle. Each powdered sample (100 mg) was extracted with 2.0 mL of water: formic acid (95:5, v/v) vortexing for 5 min, followed by sonication for 20 min at room temperature. The slurry mixture was centrifuged at 10,000 rpm at 4 °C for 15 min (IEC Clinical Centrifuge at Damon/IEC Division, Needham, MA, USA). The supernatant was filtered through a 13 mm (0.45 μm) PTFE syringe filter (Advantech DISMIC-13HP, Toyo Roshi Kaisha, Ltd., Tokyo, Japan), and 1.5 mL of the extract was used for further HPLC analysis. The HPLC system (1200 series, Agilent Technologies, Palo Alto, CA, USA) used in the study was equipped with a PDA LC detector. Individual anthocyanins within the extract solution were separated on a Synergy 4 μ Polar-RP 80A (250 × 4.6 mm, i.d.) column with a Security Guard AQ C18 (4 × 3 mm, i.d.) both purchased from Phenomenex (Torrance, CA, USA). The detection wavelength and temperature of the column oven were set at 520 nm and 40 °C, respectively. Solvent A consists of water: formic acid (95:5, v/v) and solvent B consisted of acetonitrile: formic acid (95:5, v/v). The flow rate was maintained at 1.0 mL/min and the injection volume was 10 µL. Samples were eluted with gradient condition as follows: started at 0–8 min, 5%–13% B; 8–13 min, 13% B; 13−20 min, 13%–17% B; 20–23 min, 17% B; 23–30 min, 17–20% B; 30–40 min, 20% B; 40–40.1 min, 20-5% B; and 40.1–50 min, 5% B (total 50 min) [18]. Peaks were identified and qualified using Empower 3 software (Waters Corporation, Milford, MA, USA). The samples of T1 and T2 generations of transgenic black nightshade plants were analyzed by LC/MS/MS mass spectrometry. The contents of total anthocyanins were measured by a spectrophotometric device.

### 2.3. LC/MS/MS Analysis

For the LC-MS/MS of transgenic black nightshade plants was analyzed using a Triple TOF 5600 system composed of a Hybrid Quadrupole-TOF LC/MS/MS Mass Spectrometer (AB Sciex Instruments, Framingham, MA, USA); Model 5035153/M; Serial Number BN24891607; Source Housing DuoSpray Ion Source; Sampler G7129B; Metering 40 µL Analytical Heat; DAD G1315D Serial Number DEAAX09767; Vacuum Gauge (10 × 10^−5^ Torr) at pressure 3.0 temperature 500.0 °C. The LC-MS conditions were set as follows: using the positive ion mode ([M]+) MS/MS High Resolution; duration of 3 µs; scan range from 200 to 1500 *m*/*z*; pulser frequency value of 15.392 kHz; sample acquisition duration 65 min; ion tolerance 50.000 mDa.

### 2.4. Measurement of Total Anthocyanins Content by Spectrophotometry

To determine the total anthocyanin content, 50 mg of the dry weight of the sample in 15 mL tube was extracted using 3 mL of methanol in 1% of HCl (v/v) and incubated at 4 °C to avoid light for 16 h. The extracts were centrifuged at 10,000 rpm at 4 °C for 15 min. The supernatant was transferred to a new 15 mL tube. Then the sample was diluted five times with an extraction buffer for measuring the spectrophotometric absorption at 530 nm and 650 nm. Total anthocyanin content was calculated as previously described [19].

### 2.5. Plant Growth and Optimal Kanamycin Selection Media

Leaf explants of black nightshade were tested to select the optimal concentration of kanamycin on plant regeneration medium. The various concentrations of kanamycin (10, 20, 30, 50, 100 mg/L) were used in Murashige and Skoog (MS) media supplemented with 3% sucrose and 0.8% agar that contained 2.0 mg/L of 6-benzylaminopurine (BAP) and 0.1 mg/L of (1-naphthalene acetic acid (NAA). Excised leaves within 5 mm × 5 mm size were placed on plant regeneration media and then incubated at room temperature and 25 °C under 16 h light/8 h dark condition. After 1 month approximately, no shoot was induced on medium containing 50 mg/L of kanamycin. Thus, a full range of MS supplemented with 2.0 mg/L of BAP, 0.1 mg/L of NAA, and 50 mg/L of kanamycin was used as transgenic selection media.

### 2.6. Construction of AtPAP1 Overexpression Vector

**Figure 1:**
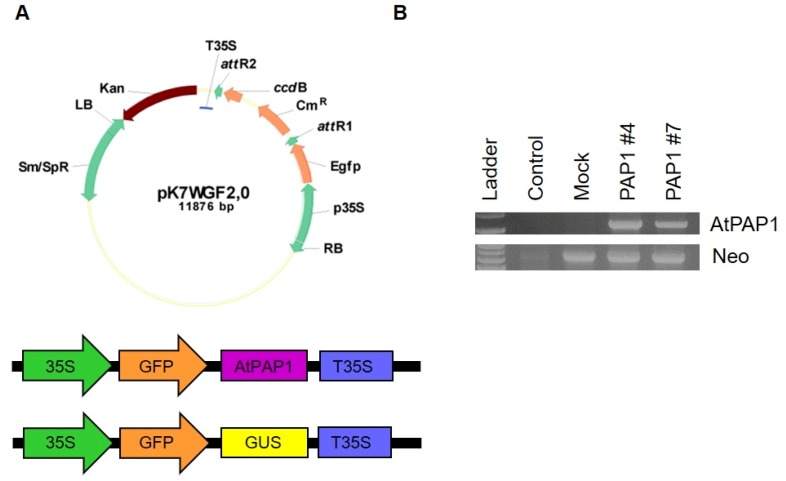
The schematic diagrams of T-DNA region of transformation vectors. (**A**) Plasmids pK7WGF2-GFP-PAP1 and pK7WGF2-GFP-GUS were used to transform *S. nigrum*. (**B**) qRT-PCR analysis of AtPAP1 gene and kanamycin selection marker (Neo) gene in AtPAP1overexpression lines.

The sequence of *AtPAP1* was clone into pDONR 221 vector using BP clonase II (Invitrogen) according to the manufacturer’s instructions. The amplified genomic coding region was transformed into TOP10 cells (Invitrogen, Carlsbad, CA, USA) and transferred to the pK7WGF2 destination vector for N-terminal GFP-fusion and C-terminal GFP-fusion (http//www.psb.ugent.be/gateway) using LR clone (Invitrogen). The resultant AtPAP1 overexpression constructs consisted of a 35S promoter, Green fluorescence protein (GFP), the respective gene sequences, and the neomycin phosphotransferase (NPTII) gene as a selectable marker (Figure 1). The pk7WGF2 plasmid was then transferred into *Agrobacterium tumefaciens* GV3101 by electroporation and grew at 28 °C on Luria-Bertani (LB) medium with rifampicin, and spectinomycin at a concentration of 50 g/mL. A pK7WGF2 vector consisted of a 35S promoter GFP-GUS fusion and the neomycin phosphotransferase (*NPTII*) gene as a selection marker was used as a control. Excised leaves of *S. nigrum* from 1-month old seedlings were used as the explant material for co-culture with into *A. tumefaciens* GV3101 harboring GUS, and AtPAP1 overexpression constructs.

### 2.7. Agrobacterium Preparation

*A. tumefaciences* GV3101:pK7wgf2: GFP: PAP1 was inoculated from glycerol stock and cultured in a 30 mL of LB (1% tryptone, 0.5% yeast extract, and 1% NaCl, pH 7.0) liquid media supplemented with 50 mg/L of gentamycin and 25 mg/L of rifampicin, following overnight incubation at 28 °C.

### 2.8. Co-Culture with Agrobacterium

*Agrobacterium* mid-log phase (O.D A600 = 0.6) cells were harvested by centrifugation at 4,000 rpm at 4 °C for 10 min. *A. tumefaciens* was resuspended in half-strength MS liquid media and cell density was adjusted to an O.D A600 of 1.0 for plant co-culture. The leaf explant from 3 weeks old seedling was wounded by using a scalpel, then immersed in *A. tumefaciens* GV3101 suspended liquid medium for 15–20 min. The leaf explants were then incubated on MS media supplemented with 2.0 mg/L of BAP and 0.1 mg/L of NAA without antibiotics for two days. The infected leaf explant was transferred to the plant regeneration media containing MS supplement with 2.0 mg/L of BAP, 0.1 mg/L of NAA, and antibiotics (50 mg/L of kanamycin + 500/250 mg/L of cefotaxime) for one week. The single shoot emerged from the wounded site was separated from the leaf explant and transferred to the rooting media containing ½ MS media supplemented with antibiotics (50 mg/L of kanamycin + 250 mg/L of cefotaxime). After 2 weeks of rooting, a single whole plant was transplanted to the pot and acclimatized for two weeks before transferring to the growth chamber.

### 2.9. PCR Analysis of the Transgenic Lines

Plant genomic DNA for polymerase chain reaction (PCR) was extracted by the Genomic DNA mini kit (Geneaid Biotech Ltd., New Taipei City, Taiwan), following the manufacturer’s instructions. The leaves of transformation (AtPAP1) lines and control (GUS and GV3101) plants were ground into a fine powder (50–100 mg fresh weight) using a mortar and pestle. Extracted genomic DNA was subjected to PCR analysis using gene-specific primers provided in Table 1. 

The PCR conditions consisted of denaturation at 95 °C for 30 s, annealing at 58 °C for 30 s and extension at 72 °C for 30 s, followed by 35 cycles and the final extension at 72 °C for 5 min. The PCR products were analyzed on a 1% agarose gel stained with ethidium bromide and visualized under UV light.

### 2.10. Transgenic Plant Seed Segregation Assay

The transgenic plants were grown in a growth chamber for four months and the mature seeds of T0 primary transgenic plants were harvested. Twelve AtPAP1 overexpression transgenic seeds (T1) were harvested from seeds germinated on half-strength MS media with 50 mg/L Kanamycin selection marker. Then five shoots of kanamycin-resistant in each line were grown on the pot (T_2_) for three months. The transgenic plants were grown in a growth chamber for four months and the mature seeds of T_0_ primary transgenic plants were harvested. Following generations (i.e., T1 and T2) screened on 50 mg/L kanamycin media were subjected to phenotype and anthocyanin analysis.

### 2.11. Total RNA Extraction and cDNA Synthesis

Total RNA was isolated from *S. nigrum* at fruit maturity stage by using RNA Mini Kit (Geneaid) according to the manufacturer’s protocol. The quantity and quality of total RNA were examined on a 1.0% agarose gel and using NanoVue Plus Spectrophotometer (GE Healthcare Life Sciences, Pittsburgh, PA, USA), respectively.

### 2.12. cDNA Synthesis and Quantitative Real-time PCR

The cDNA was synthesized from 1 µg of DNA free total RNA using ReverTra Ace-α Kit with oligo (dT) 20 primer (Toyobo, Osaka, Japan). The resulting cDNA products were used as templates for real time-PCR analysis. For all target genes, primers were designed using Primer Design software (GeneRunner.exe) and using an online program (https://www.genscript.com/ssl-bin/app/primer). Quantitative real-time PCR was performed in a BIO-RAD CFX96 Real-time PCR system (Bio-Rad Laboratories, Hercules, CA, USA) with 2X QuantiTect SYBR Green RT-PCR Master Mix, (QIAGEN, Hilden, Germany) under the condition as follows: reverse transcription at 50 °C for 30 min, PCR initial at 95 °C for 15 min, denaturation at 94 °C for 15 s, annealing at 60 °C for 30 s, extension at 72 °C for 30 s and reaction cycle was repeated for 39 cycles at extension followed by a final extension at 72 °C for 1 min. All reactions were from three biological replicates.

### 2.13. Statistical Analysis

Statistical analyses were performed using GraphPad InStat software (Graphpad Instat Software, San Diego, CA, USA). One-way ANOVA was applied for comparisons with the Turkey test. All data are expressed as mean ± SEM. The significance threshold was at a level of 5% (* *P* < 0.05; ** *P* < 0.01, *** *P* < 0.001). All the experiments were biological triplicates.

## 3. Results

### 3.1. Identification of Phenylpropanoid Biosynthesis Genes in Black Nightshade

The sequences of genes involved in the phenylpropanoid biosynthesis pathway were identified from the RNA-seq data of the black nightshade. The retrieved sequences were subject to a BLAST search to confirm and compare with the orthologs of other *Solanaceae* plants. The genes identified in black nightshade associated with general phenylpropanoid pathways were designated as *SnPAL* (accession MT032183, 712 amino acids), *SnC4H* (accession MT032184, 505 amino acids), *Sn4CL* (accession MT032185, 545 amino acid), *SnC3H* (accession MT032186, 510 amino acids), and *SnCHI* (accession MT032187, 217 amino acids). In addition, a biosynthesis gene involved in the anthocyanin pathway (e.g., anthocyanin-5-*o*-glucosyl transferase) was designated as *SnUGT75C1* (accession MT032188, 473 amino acids), and genes more closely linked to the lignin synthesis (e.g., caffeate *O*-methyltransferase (*COMT*)) and hydroxycinnamoyl-CoA shikimate (*HCT*)) were designated as *SnCOMT* (accession MT032189, 363 amino acids), and *SnHCT* (accession MT032190, 435 amino acids) (Table 1). To add perspective to the identified sequence of black nightshade, orthologs of several other *Solanaceae* plants that showed most similarity to the black nightshade genes were also compared. Except for *SnCHS*, all the deduced amino acid sequences from various *Solanaceae* plants had sequence identity from 90 to 99 percentile between orthologous genes in *Solanaceae* plants.

### 3.2. Induction of Anthocyanin by Overexpression of AtPAP1 in Black Nightshade

We used a gain-of-function approach to examine the effect of overexpression of AtPAP1 on black nightshade. To this end, we generated the transgenic black nightshade plants that harbor AtPAP1 cDNA and GUS (control) both driven by CaMV 35S (Figure 1A). The presence of the transgenes was confirmed by PCR analysis of AtPAP1 transgenic plants using Kan primers (Neo). Expected sizes of amplicons (630 bp) were observed in the corresponding *S. nigrum* plant lines (Figure 1B). In addition, the expression of *AtPAP1* transgene was verified by quantitative RT-PCR (Figure 1B). 

**Figure 2:**
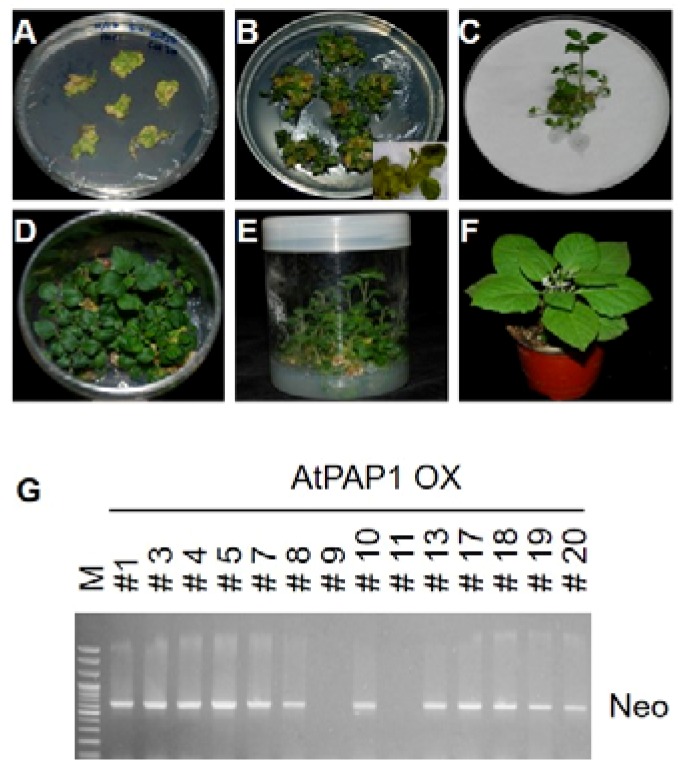
Schematic diagram of AtPAP1 overexpression transgenic lines of *S. nigrum* by Agrobacterium-mediated transformation. (**A**) Shoot regeneration for 30 days after AtPAP1 transformation. (**B**) Shoot regeneration after sub-culture for 30 days of AtPAP1 transformation. (**C**) & (**D**) Development of the whole plant. (**E**) Transgenic plant rooting. (**F**) Transgenic plant after transfer to the pot for 40 days. (**G**) Confirmation of transgene using kanamycin (Neo) primer in diverse transgenic lines.

**Figure 3:**
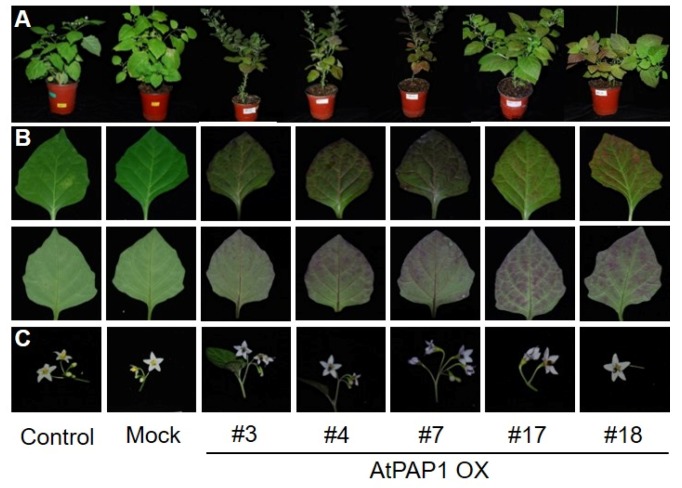
Phenotype comparison of AtPAP1 overexpression transgenic lines of *S. nigrum*. (**A**) Whole plants of AtPAP1 transgenic lines and the control line at fruit development stage in a growth chamber. (**B**) Leaf and (**C**) flower phenotype comparison between AtPAP1 overexpression lines and control (GFP-GUS (Control) and GV3101 (Mock)) lines.

More than 15 individual transgenic lines were obtained and maintained in a growth chamber (Figure 2A–F), for which the transgene was also confirmed by PCR using Kan primers (Neo) (Figure 2G). Overexpression of *Arabidopsis* AtAPAP1 resulted in enhanced color changes all over the plant including leaves and flowers compared with the control plant (Figure 3A). The appearance of purple color was especially notable in leaves and floral organs such as petals and anthers (Figure 3B,C). Thus, our data demonstrate that overexpression of a single *Arabidopsis* regulatory gene, *AtPAP1*, might have increased the content of anthocyanin in the heterologous system of black nightshade.

### 3.3. Expression of Anthocyanin Biosynthesis Genes in the AtPAP1-overexpressed Black Nightshade Plants

**Figure 4:**
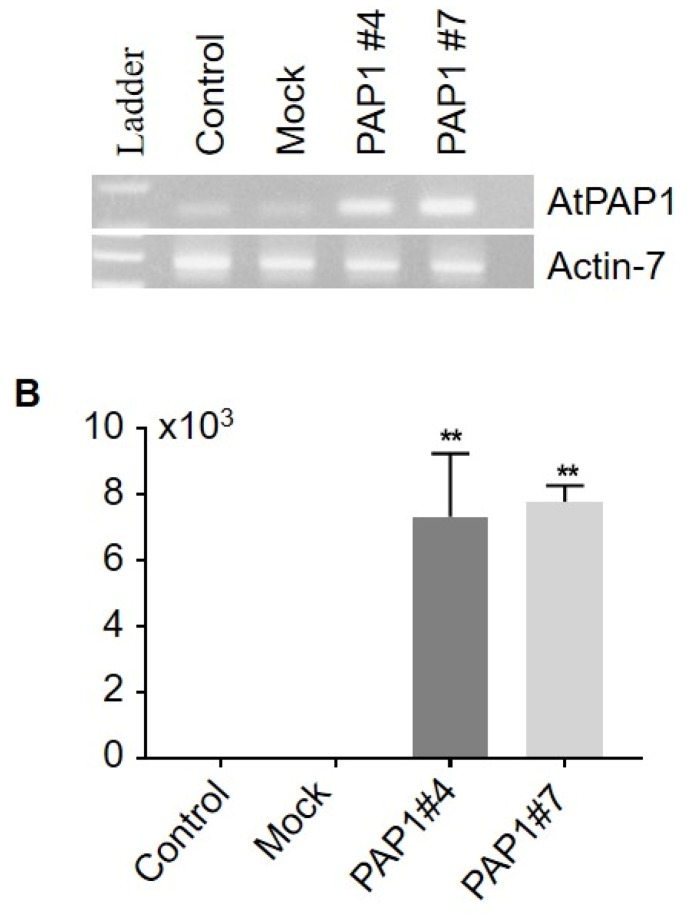
Expression of AtPAP1 gene in AtPAP1 overexpressing *S. nigrum* transgenic lines and control lines. All data are expressed as mean and SEM. The significance threshold was at a level of 5% (* *P* < 0.05; ** *P* < 0.01).

**Figure 5:**
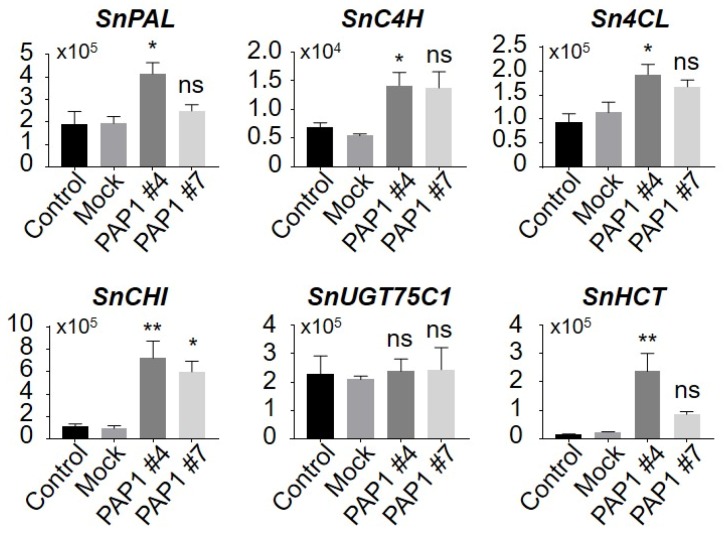
Expression of flavonoid biosynthetic genes in AtPAP1 overexpressing *S. nigrum* transgenic lines and control lines. The AtPAP1 transgenic lines were harvested and proceeded to qRT-PCR analysis. SnPAL, phenylalanine ammonia-lyase; SnC4H, cinnamate-4-hydroxylase; Sn4CL, 4-coumarate: CoA ligase; SnCHI, chalcone isomerase; SnUGT75C1, UDP-glycosyltransferase 75C1; SnHCT, quinate hydroxycinnamoyl transferase. All data are presented as mean and SEM. The significance threshold was at a level of 5% (* *P* < 0.05; ** *P* < 0.01,). All the experiments were biological triplicates.

To understand the mechanism underlying the increased content of anthocyanin in the transgenic black nightshade, we examined gene expression of anthocyanin biosynthesis that might have been affected by overexpression of AtPAP1. As expected, expression of AtPAP1 transgene was up-regulated in the transgenic black nightshade plants (PAP1 #4 and #7) compared with the control (GFP-GUS) and Mock (GV3101) lines (Figure 4). When we examined the genes identified in this study, associated with the phenylpropanoid biosynthesis pathway, most of the genes were highly induced in the AtPAP1 overexpression lines (Figure 5). Of the genes identified in the current study, genes known to be associated with general phenylpropanoid pathways (EBGs), such as *SnPAL* (accession MT032183)*, SnC4H* (accession MT032184)*, Sn4CL* (accession MT032185)*,* and *SnCHI* (accession MT032187) were all highly induced in the AtPAP1 overexpressed plants. Expression of other biosynthesis genes associated with anthocyanin (*SnUGT75C1*, accession MT032188) and lignin (*SnHCT*, accession MT032190) was largely unaltered. This suggests that black nightshade genes involved in the general phenylpropanoid biosynthesis pathway may have been regulated by the AtPAP1 transcription factor that rendered the purple color in *AtPAP1*-overexpressing *S. nigrum* plants, which ultimately led to an increase in the levels of anthocyanin in vegetative tissue and flower parts.

### 3.4. Identification of Anthocyanins in the AtPAP1-overexpressed Black Nightshade Plants

**Figure 6:**
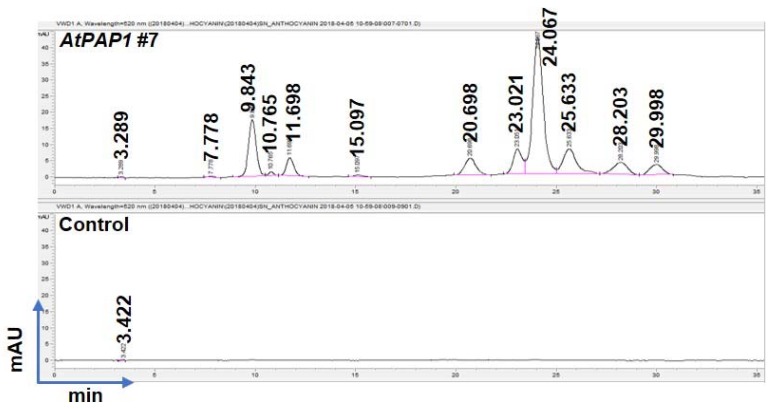
Identification of anthocyanins in AtPAP1 overexpressed *S. nigrum* transgenic lines by HPLC analysis. Above, AtPAP1 overexpression *S. nigrum* line. Detected eight peaks represented unknown anthocyanins at different retention time; Below, *S. nigrum* control line. No peak detected indicated the absence of anthocyanin.

To understand the types of anthocyanins that might have accumulated resulting from the overexpression of AtPAP1, we investigated the metabolites that correspond to anthocyanins using HPLC. From the first and second generation of AtPAP1-overexpressed transgenic black nightshade lines, we have detected several peaks associated with the various anthocyanins in black nightshade transgenic lines, while no detectable peaks have been observed in the control line (Figure 6).

Subsequent analysis of LC-MS/MS mass spectrometry has revealed that each peak of the HPLC data obtained demonstrated the same pattern of the mass ionization in both T0 and T1 transgenic lines, indicating that anthocyanins detected in two generations of transgenic plants were identical. The total ion chromatogram (TIC) was separated into 18 main peaks, from which only six peaks that represent anthocyanins were identified. The *m*/*z* ratio of each intact anthocyanin and its daughter fragments are listed in Table 2. Of these, three aglycones were determined as delphinidin aglycone (Dd, *m*/*z* 303), petunidin aglycone (Pt, *m*/*z* 317) and malvidin aglycone (Mv, *m*/*z* 331) [20]. In addition, three delphinids, two petunidin and one malvidin were detected in the AtPAP1-overexpressed black nightshade transgenic lines.

**Figure 7:**
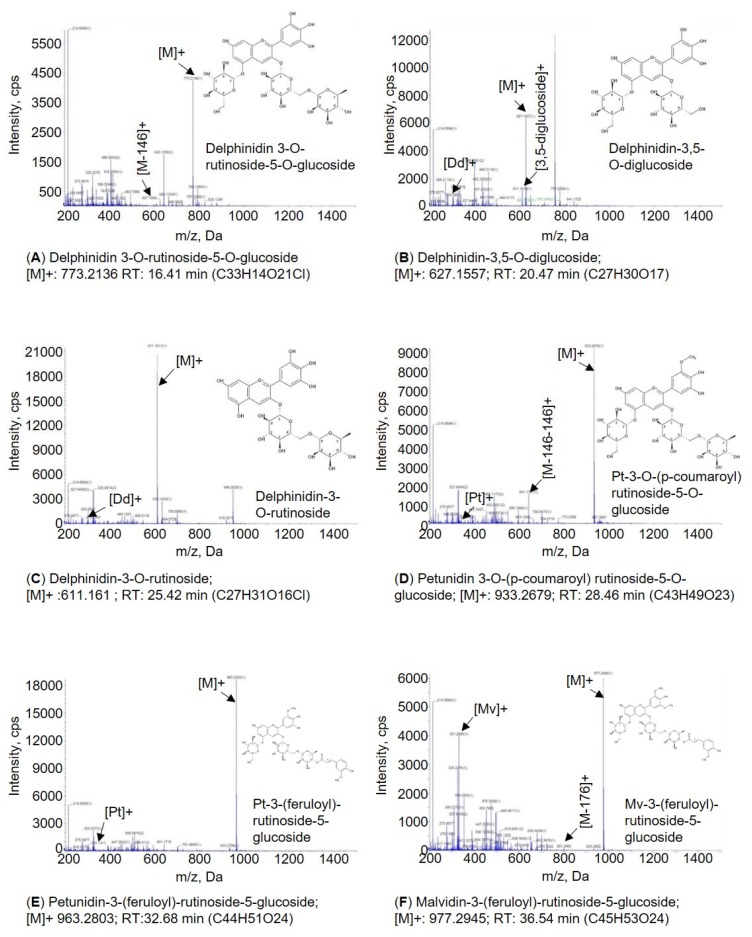
Mass spectrometric data of six anthocyanins detected in AtPAP1 overexpressed *S. nigrum* transgenic lines. (**A**) Delphinidin 3-O-rutinoside-5-O-glucoside, (**B**) Delphinidin-3,5-O-diglucoside, (**C**) Delphinidin-3-O-rutinoside, (**D**) Petunidin 3-O-(p-coumaroyl) rutinoside-5-O-glucoside, (**E**) Petunidin-3-(feruloyl)-rutinoside-5-glucoside, and (**F**) Malvidin-3-(feruloyl)-rutinoside-5-glucoside.

Specifically, the mass spectrum of peak 8 with a retention time at 16.41 min that had a molecular ion *m*/*z* of 773.2136 [M]+ and fragment *m*/*z* of 627.156 ([M-146]+) identified as delphinidin 3-O-rutinoside-5-O-glucoside with a loss of one molecule of rhamnose [21]. The peak 9 with retention time at 20.47 min that had a molecular ion *m*/*z* of 627.1557 and fragment *m*/*z* of 611.1610 was identified to be a 3,5-diglucoside glycone (m/z 303 [Dd]+ known as delphinidin aglycon), and this peak represented delphinidin-3,5-O-diglucoside. The peak 12 with a retention time at 25.42 min with *m*/*z* 611.1611[M]+ and fragmentation *m*/*z* of 464.1031 ([M-146]+) corresponds to a delphinidin aglycone that is derived from loss of one molecule of rhamnose with *m*/*z* 303 [Dp]+, was identified as an anthocyanin derivative (delphinidin 3-0-rutinoside) [21]. Peak 13 with a retention time at 28.46 min that showed a molecular ion *m*/*z* and fragment *m*/*z* of 933.2679 and 641.1721 [M-146-146], respectively, appeared as a petunidin aglycone with a loss of two molecules of coumaric acid (*m*/*z* 317([Pt]+) identified as petunidin-3-O-rutinoside (*cis*-*p*-coumaroyl)-5-O-glucoside or petunidin-3-O-rutinoside (*trans*-*p*-coumaroyl)-5-O-glucoside. The peak 15 with retention time at 32.68 min that had a molecular ion and fragment of 963.2803 and 317 [Pt]+ corresponded to a petunidin aglycone, which was tentatively identified as petunidin-3-(feruloyl)-rutinoside-5-glucoside [21,22,23]. Finally, the peak 17 with a retention time at 36.54 min was identified as malvidin-3-(feruloyl)-rutinoside-5-glucoside by the ion at *m*/*z* of 977.2945 [M]+ and the fragment at *m*/*z* of 801.2463 [M-176]+, produced by elimination of one molecule of ferulic acid (*m*/*z* 331[Mv]+) that is malvidin aglycone (Figure 7) [24].

### 3.5. Total Anthocyanin Contents induced by Overexpression of AtPAP1 in Black Nightshade

**Figure 8:**
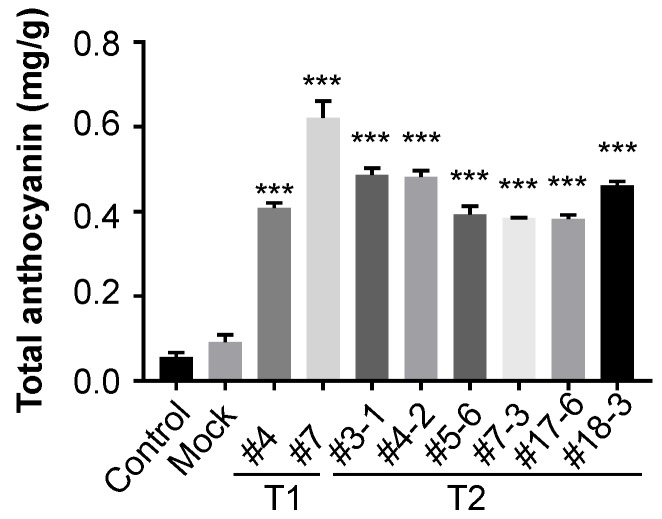
Total anthocyanin contents in AtPAP1 overexpressed *S. nigrum* transgenic lines. Total anthocyanin was measured by spectrophotometry at A530 nm and A650 nm. All data are expressed as mean and SEM. All AtPAP1 overexpressed *S. nigrum* transgenic lines from first (T1) and second generation (T2) were compared with control and mock lines. The significance threshold was at a level of 5% (*** *P* < 0.001). All the experiments were biological triplicates.

In addition to six anthocyanins identified in the AtPAP1-overexpressed transgenic black nightshade plants, we measured the total anthocyanin content in the leaves of AtPAP1-overexpressed black nightshade transgenic plants using spectrophotometry. Compared with control and mock (GV3101) lines, both first and second generation plants of the AtPAP1-overexpression lines had higher levels of total anthocyanins (Figure 8). Our data collectively demonstrate that ectopic expression of a single regulatory gene, AtPAP1, can induce total anthocyanins in black nightshade plants, likely through regulation of the conserved biosynthesis pathway of anthocyanin in black nightshade plants.

## 4. Discussion

In the current study, we have demonstrated the accumulation of anthocyanins by overexpressing an AtPAP1 in the black nightshade plant. Expression analysis of the genes associated with the biosynthesis of general phenylpropanoid and flavonoid pathways indicates that not all the regulatory genes of anthocyanins may not be transcriptional targets of AtPAP1, and may require unidentified species-specific mechanisms. Nonetheless, a single *Arabidopsis* MYB transcription factor, AtPAP1, was sufficient to enhance the accumulation of anthocyanins in a heterologous system of black nightshade.

In *Arabidopsis*, it has been shown that the biosynthesis of anthocyanins and proanthocyanidins is predominantly regulated by the MBW transcriptional complex that consists of MYB, bHLH, and WD-40 [25]. Alternatively, an *Arabidopsis* MYB transcription factor, AtPAP1, has been shown to be sufficient to promote accumulation of anthocyanin in a heterologous plant system such as dandelion (*Taraxacum brevicorniculatum*) [26]. Novel MYB transcription factors from the American plum (*Prunus americana*) have been also demonstrated to be effective in the accumulation of anthocyanins in tobacco and citrus without requiring any further transcriptional co-regulators except for *PamMYBA3*, which appears to be dependent upon an interacting protein (bHLH) to form a functional transcriptional regulatory unit [27]. Moreover, overexpression of MYB transcription factor, EsMybA1, from *Herba epimedii* (*Epimedium sagittatum)* was sufficient to induce high levels of anthocyanins in the transgenic tobacco and *Arabidopsis*, via up-regulation of major flavonoid biosynthetic pathway genes [28]. These reports indicate that some MYB transcription factors physically associate with the co-regulators such as bHLH, while other MYB transcriptional factors appear to be sufficient to induce the accumulation of anthocyanins in diverse plant species [29].

From our study, ectopic expression of an *Arabidopsis* AtPAP1 alone was sufficient to induce anthocyanin production in black nightshade, resulting in the purple-colored phenotype in both vegetative and reproductive tissues. It is noteworthy that *Gerbera* MYB transcription factor GMYB10, an *Arabidopsis* ortholog of AtPAP1, activates some of EBGs of general phenylpropanoid pathway genes (*PAL, C4H, CHI,* and *F3H*) and accumulate anthocyanins [30]. It would be interesting to know if the genes identified as EBGs of black nightshade are also the transcriptional targets of AtPAP1, which confers the pigmentation of anthocyanins in the transgenic lines.

Although numerous studies about anthocyanins in berries (e.g., cherry, blueberry, blackcurrant) have been reported to date, studies of anthocyanins in black nightshade are still scarce. Nonetheless, a recent study by Wang et al. [20] has shown that eight anthocyanins with four anthocyanin aglycones such as cyanidin (Cy), malvidin (Mv), petunidin (Pt) and delphinidin (Dp) were detected in the peel and flesh of black nightshade fruit [20]. In our study, we have identified six anthocyanins that included three delphinids, two petunidin and one malvidin from leaf tissue of AtPAP1-overexpressed transgenic black nightshade plants. Among the anthocyanins identified in this study, delphinidin 3-O-rutinoside-5-O-glucoside, delphinidin-3,5-O-diglucoside, delphinidin-3-O-rutinoside, and malvidin-3-(feruloyl)-rutinoside-5-glucoside were also detected in the fruit of black nightshade [20]. In addition to the above four anthocyanins, two petunidin, petunidin-3-O-rutinoside (*cis*-*p*-coumaroyl)-5-O-glucoside and petunidin-3-(feruloyl)-rutinoside-5-glucoside, were identified only in our study. Thus the latter two appear to be leaf-specific anthocyanins that are regulated by AtPAP1 in black nightshade.

In addition to developmental regulation, biosynthesis of anthocyanins is affected by various abiotic factors (e.g., light, sucrose, nitrogen) and phytohormones [5]. Stress tolerance against drought, high salinity, light and cold are often correlated with anthocyanin accumulation in plants [31]. These various abiotic stress conditions are known to induce a distinct set of anthocyanins, indicating that anthocyanins have different biological functions, or that coloration patterns of each anthocyanin may play a unique role in response to a certain stress factor [32]. It would be interesting to examine whether enhancing anthocyanin production in a black nightshade would help mediate stress tolerance against stress conditions. Of interest in this regard was that anthocyanin pigmentation of transgenic black nightshade plants has disappeared when grown in the greenhouse (data not are shown), which suggests that biosynthesis of anthocyanins in black nightshade governed by AtPAP1 is under the control of light and likely temperature conditions. How diverse stress conditions that would affect the accumulation of anthocyanins in black nightshade remain to be investigated.

## 5. Conclusions

In this present study, we have shown that an *Arabidopsis* MYB transcription factor, AtPAP1, is sufficient to induce the accumulation of anthocyanins in black nightshade that may provide the platform for the basic analysis of phytochemical biosynthetic genes and their final compounds. Furthermore, a black nightshade can be a model system to study AtPAP1-induced anthocyanin in response to various stress conditions. Further identification of detailed mechanisms that are associated with an AtPAP1-mediated and environment-modulated accumulation of anthocyanins remains to be investigated. Taken together, our data lay a foundation to study the molecular mechanisms of biosynthesis of flavonoid and anthocyanin pathways to improve the phytochemical properties in this species.

## Figures and Tables

**Table 1:** Comparison of phenylpropanoid biosynthetic genes of *S. nigrum* with most orthologous genes.

Genes	Length (amino acid)	Orthologous genes (Accession No.)	Identity (%)
SnPAL	712	*Solanum pennellii* PAL XP_015055648.1	97
		*Solanum lycopersicum* PAL XP_004249558.1	97
		*Capsicum baccatum* PAL PHT36452.1	96
SnC4H	505	*Solanum tuberosum* C4H ABC69046	99
		*Solanum pennellii* C4H XP_015078931.1	99
		*Capsicum baccatum* C4H PHT46927.1	97
Sn4CL	545	*Solanum tuberosum* 4CL NP_001305568.1	95
		*Solanum pennellii* 4CL XP_015070224.1	95
		*Solanum lycopersicum* 4CL NM_001346841.1	95
SnC3H	510	*Solanum tuberosum* C3H XP_006362631.1	94
		*Solanum pennellii* C3H XP_015084850.1	93
		*Solanum lycopersicum* C3H XP_004228867.1	93
SnCHS	271	*Nicotiana tabacum* CHS XP_016494186.1	81
		*Solanum tuberosum* CHS XP_006367318.1	76
		*Solanum pennellii* CHS XP_015060638.1	75
SnCHI	217	*Solanum brevicaule* CHI APZ86742.1	94
		*Solanum tuberosum* CHI APZ86744.1	93
		*Solanum melongena* CHI ANN02871.1	90
SnUGT75C1	473	*Solanum tuberosum* UGT75C1 XP_006358760.1	92
		*Solanum pennellii* UGT75C1 XP_015088519.1	91
		*Lycium barbarum* UGT75C1 BAG80544.1	86
SnCOMT	363	*Solanum tuberosum* COMT XP_015164331.1	95
		*Nicotiana attenuata* COMT OIT03318.1	93
		*Solanum pennellii* COMT XP_015070697.1	93
SnHCT	435	*Solanum pennellii* HCT XP_015070028.1	97
		*Solanum lycopersicum* HCT XP_004235891.1	96
		*Capsicum annuum* HCT NP_001311756.1	96

**Table 2:** The molecular ionization characteristic of anthocyanin detected in AtPAP1 overexpression *S. nigrum* lines.

Peak No.	RT (min)	[M]+ (m/z)	Fragmentation (*m*/*z*)	Tentative Identification	Molecular Formula
1	5.4400	465.0432	ND	Unknown	
2	6.2000	ND	ND	Unknown	
3	9.2600	ND	ND	Unknown	
4	10.4600	ND	ND	Unknown	
5	13.1181	ND	ND	Unknown	
6	13.5200	787.2294	ND	Unknown	
7	14.7600	617.0800	ND	Unknown	
8	16.4103	773.2136	627.1565 ([M-146]+)	Delphinidin 3-O-rutinoside-5-O-glucoside	C33H41O21
9	20.9300	627.1557	611.1610 ([3,5-diglucoside]+); 303.0501 [Dd]+	Delphinidin-3,5-O-diglucoside	C27H30O17
10	22.1800	627.1557	303.0501 [Dd]+	Delphinidin-3,5-O-diglucoside	
11	24.2900	741.2243	ND	Unknown	
12	26.5900	611.1611	464.10331 ([M-146]+); 303.0501 [Dd]+	Delphinidin-3-O-rutinoside	C27H31O16
13	29.5200	933.2679	641.1721 ([M-146-146]+); 317.0605 [Pt]+	Petunidin 3-O-(p-coumaroyl) rutinoside-5-O-glucoside	C43H49O23
14	31.5517	933.2191	641.1721 ([M-146-146]+)	Unknown	
15	32.6800	963.2803	316.9172 [Pt]+	Petunidin-3-(feruloyl)-rutinoside-5-glucoside	C44H51O24
16	35.0600	947.2843	493.0000, 301.0000	Unknown	
17	36.9300	977.2945	801.2463 ([M-176]+); 331.2095 [Mv]+	Malvidin-3-(feruloyl)-rutinoside-5-glucoside	C45 H53 O24
18	40.6300	447.2900	331.0000 [Mv]+	Unknown	

* Abbreviations: M, molecular; Dd, delphinidin; Pt, petunidin; Mv, malvidin. Glycone mass and tentative assignment: 331, malvidin; 317, petunidin; 303, delphinidin; 176, glucose/ ferulic acid; 146, rhamnose/coumaric acid; 611, 3,5-diglucoside.

## Supplementary Materials

Supplementary File 1
