# Supplementary material for: Accumulation of Anthocyanins through Overexpression of AtPAP1 in Solanum nigrum Lin. (Black Nightshade)
Source: Biomolecules. 2020 Feb 11;10(2):277. doi: 10.3390/biom10020277 (PMC7072430; doi:10.3390/biom10020277)
Supplement: Supplementary File 1 [file biomolecules-10-00277-s001.pdf]

Supplementary Tables

Supplementary Table 1. Oligonucleotides and PCR conditions for genotyping.

Primers	Sequence (5' to 3')	Tm (°C)	Template size (bp)
Neo- F	CTATTCGGCTATGACTGGGC	60	630
Neo- R	AATATCACGGGTAGCCAACGC		
P 35S-F2-F	TCATCCCTTACGTCAGTGGAG	60	1,016
120-AtPAP1-R	AGCTCTTACAGGAACTTGGGC		
AtPAP1-QR-F	ATGGCCAAACAACTTTGCGA	60	141
AtPAP1-QR-R	TTAATGCCCGTGTTCCATCG		

Supplementary Table 2. Oligonucleotides and PCR conditions for qRT-PCR.

Gene	Primer sequence (5' to 3')	Tm (°C)	Size (bp)
SnPAL1 QRT-F-1	GAA GAT GGT GGC AGA GTT TAG G	60	239
SnPAL1 QRT-R-1	TGG TCC TCC TAT GAG ATG TAG C		
4CL1 QRT-F	TCG AGG AAA GTT GCT GTT GGT CTT A	60	295
4CL1 QRT-R	CAC AAC CTT CCG GAG CTG AAT C		
SnC4H QRT-F	CAACAGAAGGGAGAGATCAACG	60	280
SnC4H QRT-R	TCA TGT GTG GGA CTA AGA GAG G		
SnC3H QRT-F-1	CAC CCT CCA ACT CCA CTA ATG	60	229
SnC3H QRT-R-1	TAC GTC TAC CAG CAC CAA ATG G		
SnCHS QRT-F	TCT TGC TCT GGT TTC ATG CTC C	60	207
SnCHS QRT-R	GCC CAG CCT CAT TTA GGT CAC		
SnCHI QRT-F	CGCAGGGAATAGAGGTTTGGAG	60	290
SnCHI QRT-R	TCT CTG CAT CAG TGT AGG TTC C		
SnUGT75C1 QRT-F	GTG CAA CCA CAT GTT CTC TTG GT	60	201
SnUGT75C1 QRT-R	ATC GTA TCC ATC AGA GAA TCC CG		
SnCOMT1 QRT-F	AGT GCC TCT GTA CTT CCA ATG G	60	252
SnCOMT1 QRT-R	CAC CGG AGC CAA ACT ATA AAG C		
SnHCT QRT-F	TGT GTA GGG ACG AAG ATG GAC G	60	273
SnHCT QRT-R	TGA AGT GAA GAC CAG AAG CTC C		
SnACT7 QRT-F-1	CTG GTG ATG ATG CTC CTA GAGC	60	281
SnACT7 QRT-R-1	TGT TGG CCT TAG GGT TGA GAGG		
